# Effects of bypass sugar supplementation from the close-up period through 5 days after calving on milk production, blood profiles, and health conditions in dairy cows

**DOI:** 10.5713/ab.250489

**Published:** 2025-11-25

**Authors:** Hitomi Sato, Rika Fukumori, Moeri Kondo, Junna Morimoto, Kentaro Abe, Shuichi Iki, Hideaki Hayashi, Hidetoshi Higuchi, Kyoko Chisato, Shin Oikawa

**Affiliations:** 1 School of Veterinary Medicine, Rakuno Gakuen University, Ebetsu, Japan; 2 Hokkaido Research Station, Snow Brand Seed Co., Ltd., Yubari, Japan; 3 School of Veterinary Science, Rakuno Gakuen University, Ebetsu, Japan

**Keywords:** Dairy Cow, Glucose, Milk Yield, Transition Period

## Abstract

**Objective:**

Post-ruminal supplementation of glucose may contribute to subsequent milk production and health. The objective of this study was to evaluate the effects of bypass sugar (BpS) supplementation to dairy cows during the transition period on milk production and metabolic status after calving.

**Methods:**

Fifteen Holstein cows were randomly assigned into three groups considering parity and previous milk production. The three groups corresponded to the following three treatments: a BpS group supplemented with BpS, a Gly group supplemented with dietary glycerol, and a control group with no supplementation in the basal diet from 21 days pre-partum to 5 days post-partum. Cows were sampled from 21 days pre-partum to 28 days post-partum, and milk yield and blood and health statuses were determined.

**Results:**

Milk yield was higher in the BpS group than that in the other groups. The rumen fill score was highest in the BpS group from the dry period to post-partum compared with that in the other groups. Post-partum blood glucose concentrations were higher in the BpS group than in the control group. The prevalence of hyperketonemia and blood concentrations of non-esterified fatty acids, β-hydroxylbutyrate, glucagon-like peptide-2 (GLP-2) and haptoglobin were not different among the groups. In the BpS group, blood aspartate aminotransferase concentration was lower than that in the Gly group. γ-glutamyl transpeptidase was lower than that in the control group.

**Conclusion:**

From these results, feeding BpS and Gly did not affect GLP-2 secretion and alleviate inflammation after calving, but BpS increased milk production and improved liver function.

## INTRODUCTION

During the transition period (3 weeks pre-partum to 3 weeks post-partum), dairy cows undergo physiological adaptation in response to rapid endocrine and metabolic changes associated with fetal growth, parturition, and the initiation of lactation [1,2]. Peripartum diseases and the resultant culling are significant problems from an economic and animal welfare perspective. All cows experience some degree of post-partum systemic inflammation, associated with increased disease risk and decreased milk production immediately after calving [3–5]. This inflammation is associated with increased disease risk and reduced milk production, which has led to increasing attention toward controlling postpartum inflammation through anti-inflammatory drugs and nutritional strategies [1,6,7].

The gastrointestinal tract is considered one potential source of inflammation [8], and the hypothesis is that a sudden switch to highly concentrated feed during the perinatal period increases endotoxin concentrations within the gastrointestinal tract, potentially promoting its passage into the bloodstream and contributing to systemic inflammation [9,10]. Systemic inflammation can be either acute or subclinical without obvious clinical symptoms, both of which increase the utilization of amino acids and glucose to support immune responses, thereby negatively affecting milk production [8].

The small intestine is the primary organ for both the digestion and absorption of sugars (carbohydrates) and a major site for consuming or metabolizing them [11]. Since the small intestine also requires glucose, supplying additional sugars that can bypass the rumen and be absorbed in the small intestine may support the increased glucose demand for rapid fetal growth and colostrum synthesis approximately 3 weeks before parturition (close-up period) [12]. In ruminants, including dairy cows, most dietary carbohydrates are fermented in the rumen, and glucose is supplied mainly via hepatic gluconeogenesis from propionate [13]. However, because glycogen storage in the liver and muscles is limited and feed intake after calving often falls short, protein breakdown and gluconeogenesis from amino acids such as alanine increase to meet glucose demand [4,14]. Therefore, supplementation with glucogenic precursors or readily available glucose during the transition period may help improve health and productivity.

Glycerol and propylene glycol are commonly used glucogenic supplements in dairy nutrition. Glycerol is partially fermented in the rumen into propionate and butyrate, or absorbed and metabolized via glycerol-3-phosphate to dihydroxyacetone phosphate, entering the gluconeogenic pathway [15,16]. Propylene glycol is either fermented into propionate and propanol or absorbed and converted into lactate, then pyruvate, and finally oxaloacetate for gluconeogenesis [17,18]. These compounds have been shown to reduce the incidence of postpartum ketosis and improve productivity when supplemented during the dry period [19,20]. Rumen bypass sugar (BpS) is a formulation in which sucrose escapes ruminal degradation and reaches the small intestine to increase glucose supply to the gut and peripheral tissues [21]. Previous studies have reported that glucagon-like peptide-2 (GLP-2) promotes intestinal growth and repair [22], and that sugar sources stimulate GLP-2 secretion in the small intestine [23]. Through these mechanisms, BpS is expected to increase glucose supply in the small intestine and stimulate GLP-2 secretion in dairy cows. This may contribute to intestinal epithelial repair, maintenance of tight junctions, and suppression of inflammation, ultimately improving health status and milk production. However, the effects of BpS supplementation during the transition period on GLP-2 secretion, intestinal function, milk production, and overall health have not been fully investigated. In this study, we supplemented rumen BpS or glycerol from the close-up period (3 weeks prepartum) to five days after parturition and compared the effects with an untreated control group. We examined the impacts on GLP-2 secretion, milk production, blood parameters, and health status.

## MATERIALS AND METHODS

### Animals and management

A feeding study using dairy cows was conducted at the Hokkaido Research Station Snow Brand Seed, Naganuma, Hokkaido, Japan. The cows were kept in a free barn with stanchions during the dry period and 5 days after calving. The free barns were 25.2 m^2^ (3.6×7.0 m [width×length]) of living space per section, and one or two cows were kept per section. All cows were fed the same diet. They were moved to a free stall barn for milking and followed up after 100 days post-partum. Cows were fed close-up total mixed ration (TMR) once a day at 11:00 a.m from 3 weeks pre-partum. Post-partum animals were fed lactating TMR once a day at 10:00 a.m. All cows were allowed *ad libitum* intake.

### Dietary treatment

Fifteen Holstein cows (primiparous, n = 5; multiparous, n = 10) were used in this study. Five cows were randomly assigned to each of the three treatments to cater for variability in parity and body condition score (BCS) at dry-off. The treatments were BpS, glycerol, and control, and the test supplements were fed from the close-up period (3 weeks prepartum) until 5 days post-partum. The average calving number was 2.4±1.1 (1 primiparous and 4 multiparous), 2.4±1.3 (2 primiparous and 3 multiparous), and 2.2±1.1 (2 primiparous and 3 multiparous) (mean±standard error) for the BpS, glycerol, and control groups, respectively. In the BpS group, cows received 150 g/day of LipoAktiv Glu 60 (Berg+Schmidt), a rumen-protected sugar primarily composed of sucrose, with a rumen stability of ≥70%, designed to provide glucose to the small intestine, 247 mL/day of glycerol (Glycerin 85-QB for Feed; Yuka-Sangyo), both mixed into 2 kg of TMR to ensure complete intake before offering the remaining ration. No supplements were given to the control group. Feed composition and ingredients are presented in Table 1.

### Sampling

Blood and fecal samples were collected at 10:00 a.m. on −21, −14, and −7 days relative to the expected calving date, and at 3, 6, 12, 15, 21, and 28 days in milk (DIM) after calving. For pre-partum sampling, actual calving dates were used to adjust DIM, and samples were collected within±2 days of each scheduled sampling point. For post-partum sampling, samples were collected within±1 day of each scheduled DIM. Blood samples were collected in heparin (Terumo Corporation), ethylenediaminetetraacetic acid (EDTA) (Terumo Corporation), and plain tubes (Terumo Corporation) and stored at 4°C. EDTA blood was used for blood cell fractionation measurements. Heparin and plain tubes were used for centrifugation at 3,000×g for 15 min at 4°C 3 h after sample collection to obtain plasma and serum and stored at −30°C until analysis. Fecal samples were obtained by diluting the rectal stool 1:1 with water and pH was measured using a pH meter (Thermo Fisher Scientific). The remaining samples were stored at −30°C until assayed.

Body weight (BW), BCS [24], and rumen fill score (RFS) [25] were routinely measured from dry-off until 28 days post-partum. Milk yield was recorded up to 100 DIM using a computerized milk-measuring device using the Metatron system (Orion Machinery Corporation). Milk test data were extracted from the first post-partum Dairy Herd Improvement test records. Feed and residual feed were collected weekly and dried in a 60°C ventilated dryer (Thermo Fisher Scientific). Diseases were recorded up to 30 days post-partum. Dry matter intake (DMI) was measured only in −21, −14, and −7 DIM, and averaged. If two cows were housed together, the intake was halved and used as the individual DMI.

### Laboratory analysis

Serum samples were sent to Sapporo Clinical Laboratory for biochemical analyses. Glucose (Glu), total cholesterol (T-Cho), non-esterified fatty acids (NEFA), β-hydroxybutyrate (BHBA), total protein (TP), blood urea nitrogen (BUN), albumin (Alb), calcium (Ca), aspartate aminotransferase (AST), and γ-glutamyl transpeptidase (γGTP) were measured using an automated analyzer (OLYMPUS AU680; Olympus Corporation). The assays employed were Quick Auto Neo T-Cho II (Shino-Test Corporation) for T-Cho, Albumin HR II (Fujifilm Wako Pure Chemical Corporation) for Alb, Ca-AL Type C (Sekisui Medical) for Ca, Shikarikit AST (Kanto Chemical) for AST, and Shikarikit γ-GTP (Kanto Chemical) for γGTP. Serum haptoglobin concentrations were measured by enzyme-linked immunosorbent assay (ELISA) on 3 and 28 DIM using the COW HAPTOGLOBIN ELIZA KIT, HAPT-11 (Life Diagnostics). Blood cell counts and differentials were measured on a single day each prepartum (−14 or −7 DIM) and postpartum (6, 9, or 12 DIM) using an automated hematology analyzer (Thinka CB-1010; Arkray). Plasma concentrations of insulin, insulin-like growth factor-1 (IGF-1), glucagon-like peptide-1 (GLP-1), and GLP-2 were determined using a time-resolved fluoroimmunoassay method, as described in the previous chapter. Insulin, IGF-1, GLP-1, and GLP-2 were measured following the methods reported by Masuda et al [26], Laarman et al [27], Fukumori et al [28], and Elsabagh et al [29], respectively. For fecal digestibility evaluation, a digestion analyzer (Digestion Analyzer; NASCO) was used. Colostrum Brix values were measured with a digital refractometer (Atago).

### Statistics

Milk yield data were summarized weekly from 7 to 98 days postpartum. For each weekly data point, the mean milk yield over three consecutive days (the day before, the day of, and the day after the specified day) was used. Ketosis was defined as a plasma BHBA concentration of ≥1.2 mM, and the incidence rate of ketosis was calculated as the proportion of cows that tested positive for ketosis in each treatment group [7]. Other peripartum disorders included mastitis, hypocalcemia, and retained placenta.

All data are presented as least squares means±standard error of the mean (SEM). Except for the number of diseased cows, all data were analyzed using JMP (ver. 13.0 for Windows; SAS Institute). The statistical model also included prepartum DMI, prepartum fecal digestion fraction, BW loss, BCS loss, colostrum Brix value, calf BW, milk yield, and milk composition (fat, lactose, and protein) as response variables. In this model, treatment was included as a fixed effect and cow was treated as a random effect. The mixed model for intake, DMI, fecal nutrient digestibility, colostrum composition, milk yield, BW, BCS, RFS, fecal pH, blood metabolites, metabolic hormones, haptoglobin concentrations, and hematological parameters included cow, parity, treatment, DIM, and treatment×DIM as fixed effects, with cow as a random effect. Peripartum disorders that occurred within 28 days postpartum were analyzed using the chi-square test based on cross-tabulation of treatment group and disease incidence. For comparisons among treatments on each day, slice tests were performed to compare BpS with the other two treatments. Statistical significance was declared at p<0.05, and trends were considered at p<0.10.

## RESULTS

**Figure 1:**
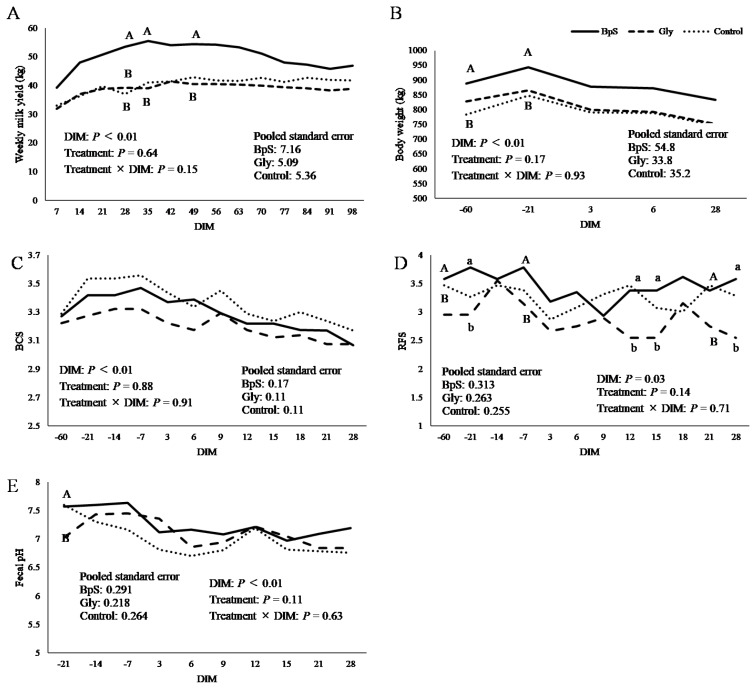
Least square means of weekly milk yield (A), body weight (B), body condition score (BCS) (C), rumen fill score (RFS) (D), and fecal pH changes (E) with DIM. Solid, dashed, and dotted lines represent BpS, Glycelol, and Control groups, respectively. ^A,B^ Means with different capital letter superscripts differ among groups (p<0.1). ^a,b^ Means with superscripts of different letters are significantly different (p<0.05). DIM, days in milk; BpS, bypass sugar.

DMI and fecal digestion fraction during the close-up period, as well as calf birth weight, BW loss, BCS loss, and milk production performance, are presented in Table 2. No significant differences were observed for all the parameters. The prevalence of post-partum diseases is presented in Table 3. Serum BHBA concentration of ≥1.2 mM was considered hyperketonemia. The prevalence of hyperketonemia had no significant difference from 3 to 28 DIM. The disease incidence also had no significant difference within 28 DIM. The milk yield (Figure 1A), BW (Figure 1B), BCS (Figure 1C), RFS (Figure 1D), and fecal pH (Figure 1E) are presented in Figure 1. Milk yield was higher in the BpS group at 28 DIM than that in the glycerol and control groups (p<0.1). At 35 and 49 DIM, the BpS group had a higher milk yield than that of the glycerol group (p<0.1). BW was significantly higher in the BpS group than in the control group at −60 and −20 DIM (p<0.1). BCS had no significant difference among the different groups. RFS was significantly higher in the BpS group than that in the glycerol group at −60, −7, and 21 DIM (p<0.1). RFS was significantly longer in the BpS group than that in the glycerol group at −21, −12, −15, and −28 DIM (p<0.05). Fecal pH was significantly higher in the BpS group than that in the glycerol group at −21 DIM (p<0.1).

**Figure 2:**
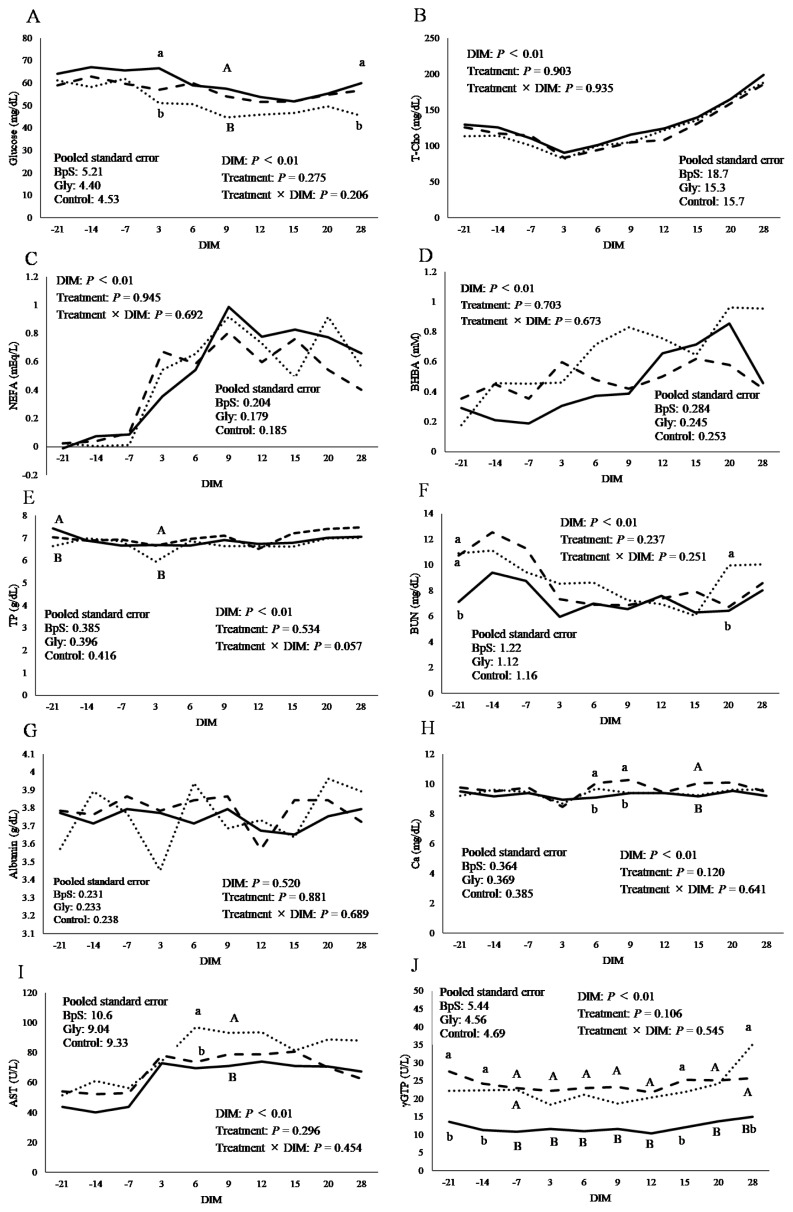
Least square means of glucose (A), total cholesterol (T-Cho) (B), NEFA (C), BHBA (D), total protein (TP) (E), BUN (F), Albumin (G), Ca (H), AST (I), and γGTP (J) with DIM. Solid, dashed, and dotted lines represent BpS, Glycerol, and control groups, respectively. ^A,B^ Means with different capital superscript letters are different among groups (p<0.1). ^a,b^ Means with different superscript letters are significantly different among groups (p<0.05). BpS, bypass sugar; DIM, days in milk, NEFA, non-esterified fatty acids; BHBA, β-hydroxybutyrate; BUN, blood urea nitrogen; AST, aspartate aminotransferase; γGTP, γ-glutamyl transpeptidase.

Serum concentrations of the metabolites are presented in Figure 2. Serum glucose concentration (Figure 2A) was higher in the BpS group than that in the control group at 9 DIM (p<0.1) and was significantly higher in the BpS group than that in the control group at 3 and 28 DIM (p<0.05). No significant differences were observed in the serum T-Cho (Figure 2B), NEFA (Figure 2C), and BHBA (Figure 2D) concentrations among the different groups. Serum TP (Figure 2E) concentration was higher in the BpS group than that in the control group at −21 and 3 DIM (p<0.1). BUN concentration (Figure 2F) was significantly lower in the BpS group than those in the glycerol and control group at −21 DIM (p<0.05) and significantly lower in the BpS group than that in the control group at −20 DIM (p<0.05). The serum Alb concentration (Figure 2G) was not significantly different among the groups. Blood Ca concentration (Figure 2H) was lower in the BpS group than that in the glycerol group at 15 DIM (p<0.1), and was significantly lower in the BpS group than that in the glycerol group at 6 and 9 DIM (p<0.05). Serum AST concentration (Figure 2I) was lower in the BpS group than that in the control group at 9 DIM (p<0.1) and was significantly lower in the BpS group than that in the control group at 6 DIM (p<0.05). Serum γGTP concentration (Figure 2J) in the BpS group was lower than those of the glycerol and control groups at DIM −7 (p<0.1), was lower in BpS group than that in the glycerol group at −3, 6, 9, 12, 20, and 28 DIM (p<0.1), significantly lower than that in the glycerol group at −21 and −14 DIM (p<0.05), and significantly lower than that in control group at −28 DIM (p<0.05).

**Figure 3:**
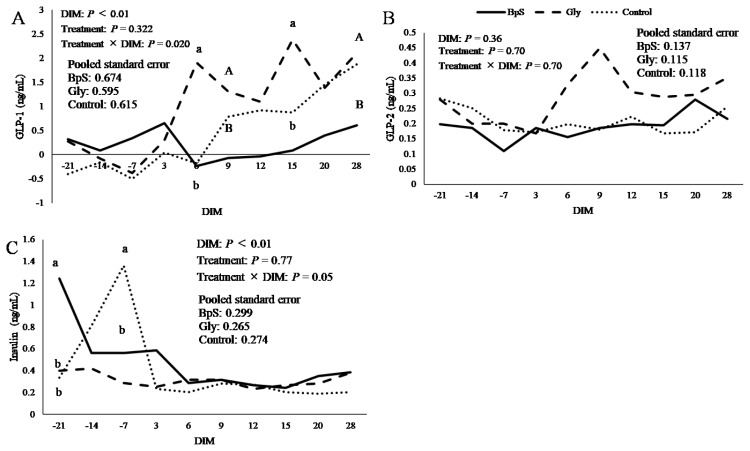
Least square means of glucagon-like peptide 1 (GLP-1) (A), glucagon-like peptide 2 (GLP-2) (B), and insulin (C) with DIM. Solid, dashed, and dotted lines represent BpS, Glycerol, and control groups, respectively. ^A,B^ Means with different capital superscript letters among groups are different (p<0.1). ^a,b^ Means with different superscript letters are significantly different among groups (p<0.05). DIM, days in milk; BpS, bypass sugar.

The plasma concentrations of metabolic hormones are presented in Figure 3. Plasma GLP-1 concentration (Figure 3A) was higher in the glycerol group than that in the BpS group at 9 and 28 DIM (p<0.1) and was significantly higher in the glycerol group than that in the BpS group at 6 and 15 DIM (p<0.05). The plasma GLP-2 concentration (Figure 3B) was not significantly different among the groups. Blood insulin concentration (Figure 3C) was significantly higher in the BpS group than that in the other groups at −21 DIM (p<0.05), and significantly lower in the BpS group than that in the control group at −7 DIM (p<0.05).

Serum haptoglobin concentrations at 3 and 21 DIM were not different between treatments (p = 0.723, Table 4). Significant treatment effects were not observed in pre- and post-partum blood cell count and fraction (p≥0.362, Table 5).

## DISCUSSION

There were no significant differences in prepartum DMI, fecal properties, or post-partum performance. However, there was a higher milk yield in the BpS group than that in the other groups considering DIM. In ruminants, dietary carbohydrates are only marginally absorbed through the small intestine as glucose; however, the small intestine is the organ through which cells utilize glucose as an energy source [12]. Systemic inflammation includes acute inflammation without clinical symptoms or chronic inflammation. Both consume more amino acids and glucose to cope with inflammation, thereby reducing milk production [8]. Zhang et al [30] reported that feeding rumen bypass glucose (RPG) during the parturition transition downregulated the expression of toll-like receptor genes, such as TLR4 and inflammatory cytokines in the cecum, increased occludin expression, a tight junction protein, and enhanced mucosal immunity. They fed 200 g/d RPG to dairy cows, whereas in the present study, 150 g/d BpS was supplemented; both feeds provided 90 g of sugar per cow per day. In the present study, inflammatory status was assessed using serum haptoglobin concentration and white blood cell count, but no differences were observed among treatment groups. Because these markers do not specifically reflect intestinal inflammation or its local effects. Therefore, further evaluation using analyses of inflammatory cytokines and tight junction proteins in the small intestine is warranted. Glycerol supplementation did not show any significant effects on milk yield or metabolic indicators compared with the control group. Previous studies have reported improved milk production and energy metabolism in dairy cows supplemented with glycerol during the transition period. In the previous study by McWilliams et al [31], cows received 250 g/day of glycerol for 21 days (approximately 5.25 kg in total) [31]. Van Soest et al [32] provided 165 g/day for 42 days (6.93 kg in total) [32]. In the present study, cows were supplemented with 247 mL/day of glycerol (equivalent to approximately 210 g/day of pure glycerol) for 26 days, totaling about 5.46 kg. Thus, the total amount of glycerol supplied was comparable to previous studies; however, the feeding period (from three weeks prepartum to five days postpartum, approximately 26 days) was shorter than that reported by Van Soest et al [32] and similar to McWilliams et al [31]. Therefore, the limited feeding duration or timing may have influenced the lack of observable effects in the present study, suggesting that further research with extended supplementation periods is warranted.

Differences in serum glucose, insulin, hepatic metabolic load, and protein utilization may have contributed to the higher milk yield observed in the BpS group. Although both BpS and glycerol supplementation provided an equivalent additional energy supply of 0.9 Mcal, only the BpS group exhibited an increase in milk yield. This suggests that sugar absorbed from the small intestine in the BpS group may have been preferentially utilized for lactose synthesis, supporting enhanced milk production. In the BpS group, serum glucose concentrations were higher at 3 DIM, possibly because of sugar source supplementation from the intestinal tract. Although serum glucose concentrations were elevated, no substantial differences in post-partum plasma insulin concentrations were observed, indicating potential glucose allocation to the mammary gland. Because glucose is the primary precursor for lactose synthesis, this allocation may have contributed to increased milk yield. These metabolic responses imply that BpS positively influenced glucose metabolism in a way that supported milk production. Moreover, since the increase in milk yield occurred without a deterioration in energy balance, it is possible that BpS improved glucose utilization efficiency in the mammary gland. Although decreased insulin sensitivity is considered a risk factor for metabolic disorders during the transition period, no significant differences were observed in the incidence of hyperketonemia or other post-partum diseases. In contrast, in the glycerol group, glycerol was likely fermented in the rumen to produce propionate, which serves as a substrate for gluconeogenesis and may have elevated serum glucose concentrations [15,16]. However, such increases were not confirmed in this study. Further studies are needed to clarify the metabolic pathways involved. AST and γGTP concentrations remained lower in the BpS group. Glycogenesis occurs in the liver, and consumes adenosine triphosphate (ATP); thus, increased ATP consumption may impair hepatic metabolic function. In the BpS group, direct sugar absorption from the lower gastrointestinal tract may have reduced hepatic gluconeogenesis and energy cost, increasing glucose availability for milk production. Serum TP concentration is influenced by both Alb and globulin levels. Although no significant differences were observed among the groups, Alb concentrations at 3 DIM were numerically lower in the control group, which was consistent with the TP results. BUN reflects both feed intake and dietary protein content. RFS reflects feeding status within approximately 12 hours, and the higher RFS values in the BpS group suggest greater feed intake compared to the other treatment groups. However, as DMI postpartum was not measured in the present study, the exact cause of this difference remains unclear. At 20 DIM, BUN concentrations were significantly lower in the BpS group than in the control group. This may indicate more efficient protein utilization and reduced nitrogen excretion in the BpS group. Mg, Ca, and P concentrations were lower after post-parturition in the BpS group, suggesting that BpS supplementation did not improve mineral metabolism.

Plasma GLP-1 concentration was lower in the BpS group than that in the glycerol group, but post-partum plasma GLP-1 levels increased with DIM, which is consistent with a previous report by Relling and Reynolds [33]. Plasma GLP-2 concentrations did not differ among the groups. GLP-1/2 secretion patterns suggest that sugar absorption site may influence hormonal responses, with glycerol fermentation stimulating L cells more effectively.

Based on these changes in metabolic components, we explained why BpS supported increased milk production; however, differences in initial BW and the absence of post-partum DMI data limit full interpretation of milk yield differences. Although cows with higher BW at calving may have greater energy reserves, they may also experience greater BW loss if milk production is high, due to increased energy demands, but no significant differences were observed in the percentage of BW loss between treatment groups from DIM 3 to DIM 28, despite the BpS group showing higher milk yield. For energy balance after calving, pre-parturient blood NEFA concentrations were normal in all groups and did not indicate a low-energy state, suggesting that the overall disease incidence was low. Post-partum serum BHBA concentration was significantly elevated in all treatment groups, but there were no significant differences among the groups. Although no significant differences were detected, BHBA concentrations tended to be lower in the BpS group compared to the other groups throughout the study period. In addition, the effect of the treatments on disease incidence after parturition was not significantly different. Therefore, BpS may have been able to increase milk yield without compromising energy balance or health.

## CONCLUSION

In conclusion, BpS supplementation from the close-up period to 5 days after calving did not affect GLP-2 secretion, or overall health status such as ketosis, but showed potential to improve milk yield as an energy supplement.

## Figures and Tables

**Table 1:** Chemical composition of feed ingredients used in TMR for transition and lactating dairy cows

	Close-up TMR	Lactating TMR
Grass silage	19.5	21.1
Corn silage	26.8	28
Wheat straw	8.4	0
Wood material feed	11.2	0
Steam flaked corn	0	16
Dry concentrate mix	33.1	0
Lactating concentrate mix	0	32.2
Calcium carbonate	0.5	1.2
Sodium bicarbonate	0	1.2
Mixed supplements	0.5	0.3
DM (%)	47.5	45.3
CP (% DM)	14.5	15.3
Ether extract (% DM)	3.1	4.6
NDF (% DM)	45.7	35.3
ADF (% DM)	38.4	20.9
NFC (% DM)	28	36.9
Starch (% DM)	16	26.9
Crude ash (% DM)	8.7	7.9
ME (Mcal/kg DM)^1)^	2.41	2.75

1) Estimated by NRC [34].

TMR, total mixed ration; DM, dry matter; CP, crude protein; NDF, neutral detergent fiber; ADF, acid detergent fiber; NFC, non fiber carbohydrate; ME, metabolizable energy.

**Table 2:** Prepartum dry matter intake, fecal digestion fraction, calf body weight (BW), BW loss, BCS loss, and milk production performance

Item	Treatment			SEM	p-value

	BpS	Glycerol	Control		
Precalving dry matter intake (kg/d)^1)^	18.9	16.0	17.4	2.13	0.91
Prepartum fecal digestion fraction^2)^					
Upper (rough) (%)	42.1	42.1	43.2	0.83	0.47
Middle (%)	27.9	27.7	27.2	0.27	0.14
Low (fine) (%)	30.0	30.1	29.5	0.82	0.86
BW loss (%)^3)^	4.47	6.86	7.43	2.11	0.71
BCS loss (%)^3)^	7.63	5.41	8.41	3.47	0.75
Colostrum Brix (%)	25.4	27.0	22.4	2.05	0.20
Calf BW (kg)	43.5	51.5	48.0	5.91	0.31
Milk yield (kg)^4)^	47.0	37.8	36.9	5.45	0.33
Fat (%)^4)^	4.49	4.60	4.61	0.38	0.97
Lactose (%)^4)^	4.57	4.69	4.60	0.08	0.41
Protein (%)^4)^	3.34	3.40	3.43	0.17	0.91

1) Dry matter intake was measured at −21, −14, and −7 days relative to the predicted calving date and summarized as the mean.

2) Measured by digestion analyzer once during the dry period.

3) The percentage of BW loss and BCS between DIM 3 and DIM 28 was calculated.

4) Data were obtained from the first Dairy Herd Performance Test after calving, conducted by the Hokkaido Dairy Milk Recording & Testing Association.

BCS, body condition score; BpS, bypass sugar group; SEM, standard error of the mean; DIM, days in milk.

**Table 3:** Prevalence of postpartum diseases within 28 DIM

	Treatment			p-value

	BpS	Glycerol	Control	
HYK^1)^				
3 DIM	0% (0/5)	0% (0/5)	0% (0/5)	-
6 DIM	0% (0/5)	0% (0/5)	25% (1/4)	0.26
9 DIM	0% (0/5)	0% (0/5)	25% (1/4)	0.26
12 DIM	20% (1/5)	0% (0/4)	20% (1/5)	0.63
15 DIM	25% (1/4)	20% (1/5)	0% (0/4)	0.58
20 DIM	20% (1/5)	0% (0/5)	20% (1/5)	0.51
28 DIM	20% (1/5)	0% (0/5)	20% (1/5)	0.56
Any clinical diseases^2)^	60% (3/5)	40% (2/5)	40% (2/5)	0.77

1) Hyperketonemia (BHBA>1.2 mM).

2) Mastitis, hypocalcemia, and retained placenta.

DIM, days in milk; BpS, bypass sugar group; BHBA, β-hydroxybutyrate.

**Table 4:** Serum haptoglobin concentration

DIM	Treatment (μg/mL)			SEM	p-value		
	
	BpS	Gly	Control		Treatment	DIM	Treatment×DIM
3	527	166	183	175	0.723	0.31	0.15
28	44.1	273	177	176	-	-	-

DIM, days in milk; BpS, bypass sugar group; SEM, standard error of the mean.

**Table 5:** Hematological parameters, including leukocyte count and differential, erythrocyte indices, and platelet count, before and after parturition

Item	Treatment				SEM	p-value		
	
		BpS	Glycerol	Control		Treatment	Pre- vs. post-partum	Treatment× pre-postpartum
WBC×10^9^ (/L)	Pre	9.79	12.2	9.18	2.29	0.95	0.09	0.43
	Post	7.36	6.53	8.6	2.29			
Lymph×10^9^ (/L)	Pre	3.29	5.36	3.16	1.39	0.89	0.36	0.32
	Post	3.04	2.49	3.66	0.18			
Mon×10^9^ (/L)	Pre	0.79	0.96	0.53	0.18	0.75	0.05	0.23
	Post	0.42	0.39	0.53	0.18			
Gran×10^9^ (/L)	Pre	5.8	5.9	5.49	0.94	0.99	0.03	0.79
	Post	3.9	3.65	4.41	0.94			
Lymph (%)	Pre	33.06	37.82	34.36	5.80	0.94	0.22	0.86
	Post	40.32	40	41.44	5.80			
Mon (%)	Pre	7.42	8.52	6.12	0.84	0.70	0.02	0.04
	Post	6.28	5.96	6.5	0.84			
Gran (%)	Pre	59.52	53.66	59.52	5.91	0.92	0.33	0.73
	Post	53.4	54.04	52.06	5.91			
RBC×10^12^ (/L)	Pre	6.41	6.74	6.15	0.34	0.36	0.07	0.30
	Post	5.68	6.51	6.08	0.34			
HGB (g/L)	Pre	123	117	112	4.61	0.56	0.28	0.27
	Post	110	117	113	4.61			
HCT (%)	Pre	35.22	33.42	32.28	1.51	0.75	0.40	0.15
	Post	31.32	34.24	32.72	1.51			
MCV (fL)	Pre	55.04	50.04	52.84	2.21	0.54	0.05	0.18
	Post	55.24	53.4	53.8	2.21			
RDW (%)	Pre	16.85	15.82	15.88	0.58	0.58	0.92	0.23
	Post	16.43	15.71	16.36	0.60			
PLT×10^9^ (/L)	Pre	287	310	245	57.8	0.74	0.15	0.47
	Post	262	162	214	57.8			

Pre: measured on DIM −14 or −7, Post: collected once between DIM 3 and 12.

The interaction effect was significant for Mon (%) because pre- and post-partum decrease was observed only in Gly group.

BpS, bypass sugar group; SEM, standard error of the mean; WBC, white blood cell; RBC, red blood cell; HGB, hemoglobin; HCT, hematocrit; MCV, mean corpuscular volume; RDW, red cell distribution width; PLT, platelet; DIM, days in milk.

## Data Availability

Upon reasonable request, the datasets of this study can be available from the corresponding author.
